# The association between the rs4987105 of 5-lipoxygenase (ALOX5) gene and gestational glucose metabolism in Chinese population

**DOI:** 10.1186/s13104-020-04953-2

**Published:** 2020-02-24

**Authors:** Xi Li, Jindi Su, Shiguo Chen, Sheng Lin, Xiujie Zheng, Baojiang Wang, Keqin Yao, Liping Lai, Shan Duan

**Affiliations:** 1 Laboratory of Medical Genetics, Shenzhen Health Development Research Center, 4009 Xinzhou Road, Futian District, Shenzhen, 518040 China; 2 Endocrine Department, Futian Center for Chronic Disease Control, 9 Xinsha Road, Futian District, Shenzhen, China

**Keywords:** Gestational diabetes mellitus, Genetic susceptibility, ALOX5, Polymorphism, Fasting plasma glucose

## Abstract

**Objective:**

The arachidonate 5-lipoxygenase (ALOX5) pathway has been investigated in diverse chronic inflammatory diseases including metabolic disorders. Recently, the ALOX5 polymorphism rs4987105 was identified to confer susceptibility to type 2 diabetes mellitus (T2DM), implicating its role in regulating glucose homeostasis. Gestational diabetes mellitus (GDM) shares similar pathogenic mechanism with T2DM. Thus, we aimed to evaluate the association between rs4987105 and gestational glucose metabolism in Chinese pregnant women.

**Results:**

A total of 380 unrelated Chinese pregnant women including 241 GDM patients and 139 controls were included in this study. The genotypes of rs4987105 were examined by the Agena MassARRAY iPLEX platform, the association between rs4987105 and fasting plasma glucose (FPG) levels at 24–28 gestational weeks was evaluated using different statistical methods. We found that carriers of rs4987105 CT/TT genotypes exhibited significantly lower FPG levels (*P* = 0.011). In addition, we observed a significant association between rs4987105 and FPG levels after adjusting confounding variables in the linear regression analysis using dominant genetic model (*b* = − 0.218; *P *= 0.01). The present study for the first time reported that the rs4987105 of 5-lipoxygenase (ALOX5) gene was associated with gestational glucose metabolism in Chinese pregnant women.

## Introduction

Gestational diabetes mellitus (GDM) is defined as abnormal glucose intolerance with onset or first recognition during pregnancy. It is a prevalent and clinically significant disease threatening both the mother and the offspring [1–4]. The incidence of GDM was estimated to be 8.9–53.4% worldwide and is rapidly increasing each year [5]. GDM not only brings about adverse pregnant outcomes such as stillbirth, macrosomia, shoulder dystocia, neonatal hypoglycemia and neonatal respiratory distress syndrome [6, 7], but also has substantial long-term negative effects on the health of both the patients and their offspring including increased risk of developing type 2 diabetes mellitus (T2DM), obesity, metabolic syndromes and cardiovascular diseases in later life [8, 9]. Therefore, developing effective early detection and intervention strategies to protect pregnant women against maternal and fetal complications has become an urgent need.

Normal pregnancy is associated with an altered inflammatory profile compared to the non-pregnant state. Tightly controlled balance between pro- and anti-inflammatory cytokines is necessary for normal implantation, trophoblast invasion and placentation [10–12]. Dysregulation of the immune system favoring pro-inflammatory responses has been identified as a pivotal pathogenic factor in GDM [13]. Growing evidence suggests that this enhanced degree of low-grade systemic inflammation is an important factor leading to the insulin secretion defects and uncompensated insulin resistance underlying impaired gestational glucose metabolism [13–15].

*ALOX5* encodes the central enzyme in proinflammatory leukotriene biosynthesis [16]. The lipid mediator leukotriene with increased production during inflammation plays an essential role in the development of insulin resistance and mediates β-cell destruction resulting in reduced insulin production [14, 17, 18]. Stimulated 5-lipoxygenase pathway in activated macrophages of obese mice contributed to the insulin resistant state [19]. Additionally, ALOX5 deficient mice had decreased insulin secretion and significantly increased fasting glucose levels and fat mass [20]. siRNA-mediated knockdown of *ALOX5* in isolated human islets reduced both insulin gene expression and secretion [20]. These studies underline the important role of ALOX5 in regulating glucose homeostasis.

ALOX5 and its polymorphisms have been explored as first-line candidates in a wide variety of inflammatory diseases including metabolic disorders, asthma,cardiovascular diseases, neurodegeneration, and cancer [16, 17, 20–28]. A very recent study by Nejatian et al. for the first time identified a significant association between the ALOX5 polymorphism rs4987105 and T2DM susceptibility in German population [29]. Additionally, Tsekmekidou et al. demonstrated that another ALOX5 polymorphism rs11239524 was also associated with T2DM in Greek population [30]. Since T2DM and GDM have similar pathogenic mechanism [31, 32], we speculate that polymorphisms of *ALOX5* may also have an impact on gestational glucose control. Thus, in this study we aimed to investigate the association between the more frequently studied *ALOX5* polymorphism rs4987105 and gestational glucose metabolism in Chinese pregnant women.

## Main text

### Methods

#### Subjects

The unrelated Chinese pregnant women enrolled in this study were recruited from Buji people’s hospital and Songgang people’s hospital, Shenzhen, China, between July 2012 and May 2013. The diagnosis of GDM was made based on the one-step GDM screening: a 2 h 75 g oral glucose tolerance test (OGTT) at 24–28 gestational weeks, and we adopted the International Association of the Diabetes and Pregnancy Study Groups (IADPSG) criteria: fasting plasma glucose (FPG) ≥ 5.1 mmol/L, or 1 h plasma glucose (1 h-PG) ≥ 10.0 mmol/L, or 2 h plasma glucose (2 h-PG) ≥ 8.5 mmol/L [33]. We firstly recruited 241 subjects diagnosed with GDM into the patient group, and then later we randomly collected another 139 pregnant women with normal glucose tolerance admitted to the hospitals at the same time period as the control group. A fasting plasma glucose level and 1-, 2-h OGTT plasma glucose level less than 5.1, 10.0 and 8.5 mmol/L, respectively, were defined as normal glucose tolerance. Subjects meeting the following criteria were excluded in our study: (1) the pregnant women had a past medical history of diabetes; (2) the pregnant women had a family history of diabetes or hypertension; (3) the pregnant women did not have a past medical history of diabetes but in their first antenatal examination they were diagnosed as pre-gestational diabetes mellitus (PGDM).

#### Clinical and biochemical measurements

Anthropometric variables including height, weight before pregnancy were recorded at 24–28 weeks of gestation. Pre-pregnancy body mass index (pre-BMI) (kg/m^2^) was calculated from these data. Other clinical information, such as age, educational background and family history of hypertension and diabetes, was also collected. The plasma glucose concentrations were measured with the glucose oxidase–peroxidase method using an automated biochemical instrument (Beckman Coulter, AU5800, Indianapolis, Indiana, USA).

#### Genotype analysis

Genomic DNA was isolated from peripheral blood leukocytes using QIAamp DNA Blood Mini Kit (Qiagen, Germany). The polymorphism was genotyped using the Agena MassARRAY iPLEX platform (Agena Inc., CA). For quality control we randomly tested a 5% sample of cases and controls using the same sets of primers, and the results were 100% consistent.

#### Statistical analysis

Hardy–Weinberg equilibrium (HWE) for each SNP was examined by Chi square test and there were no deviations for all genotyped SNPs in both cases and controls. Differences in clinical characteristics between GDM and control group were tested using independent sample *t*-test, Chi square test or Fisher’s exact test. The association between rs4987105 and FPG levels was analyzed using independent sample *t*-test and multivariate linear regression in the dominant genetic model with confounding factors adjusted and regression coefficients (*b*) with 95% CI presented. Pre-pregnancy BMI (kg/m^2^), maternal age at delivery (years), parity (nulliparous, multiparous), educational levels (high school or lower, college or higher), history of delivering infants with respiratory distress syndrome (yes, no), and history of macrosomia delivery (yes, no) were selected as the confounding factors adjusted in the final models based on their biologic plausibility reported in previous studies. All of the statistical tests were performed with SPSS 16.0 software and were two-sided. *P *< 0.05 was considered statistically significant.

### Results

#### Clinical characteristics of the study population

The selected characteristics of 380 participants are presented in Table 1. GDM patients exhibited significantly higher pre-pregnancy BMI (22.15 vs. 21.69, kg/m^2^) and fasting plasma glucose levels (5.18 vs. 4.31, mmol/L) than healthy controls. No statistically significant differences were observed in all other listed clinical characteristics between the two groups (Table 1).

**Table 1:** Clinical and Biomedical Characteristics of the Study Population

Characteristics	All participants (*n *= 380)	Cases (*n *= 241)	Controls (*n *= 139)	*P* value
Gestational Age (year)	28.55 ± 4.96	28.52 ± 5.06	28.59 ± 4.80	0.899
Pre-pregnancy BMI (kg/m^2^)	21.98 ± 1.95	22.15 ± 1.56	21.69 ± 2.47	*0.047*
FPG (mmol/L)	4.86 ± 0.80	5.18 ± 0.72	4.31 ± 0.62	*< 10*^*−6*^
History of delivering infants with RDS				0.366
Yes	1 (0.003%)	0 (0%)	1 (0.01%)	
No	379 (0.997%)	241 (100%)	138 (0.99%)	
History of macrosomia delivery				0.531
Yes	5 (1.3%)	2 (0.01%)	3 (0.02%)	
No	375 (98.7%)	239 (0.99%)	136 (0.98%)	
Parity				0.594
0	178 (46.84%)	110 (45.64%)	68 (48.92%)	
≥ 1	202 (53.16%)	131 (54.36%)	71 (51.08%)	
Educational level				0.363
High school or lower	344 (90.53%)	221 (91.70%)	123 (88.49%)	
College or higher	36 (9.47%)	20 (8.30%)	16 (11.51%)	

Data were presented as *n* (%) and Mean ± SD

*BMI* body mass index, *FPG* fasting plasma glucose, *RDS* respiratory distress syndrome

The significant *P* values (*P *< 0.05) are highlighted in italic

#### Association between rs4987105 and fasting plasma glucose level

We found that carriers of rs4987105 CT/TT genotypes exhibited significantly lower FPG levels (*P* = 0.011, Table 2). We also examined the association between rs4987105 and FPG levels using multivariate linear regression analysis. In order to enhance the statistical power, we combined the rare homozygous genotype with heterozygous genotype to compare with the wild-type genotype in the dominant genetic model. We observed that rs4987105 was significantly associated with this trait after adjusting confounding variables [*b* = − 0.218 (95%CI − 0.384, − 0.053); *P* = 0.01] (Table 3).

**Table 2:** Correlations Between Genotypes of rs4987105 and FPG Levels

Genotypes	Fasting plasma glucose	
	Mean ± SD	*P* Value
rs4987105		
CC	4.94 ± 0.83	
CT/TT	4.73 ± 0.74	*0.011*

The significant *P* values (*P *< 0.05) are highlighted in italic

**Table 3:** The Association Between rs4987105 and FPG Levels

SNP ID	Minor allele		Fasting plasma glucose
rs4987105	T	*b* (95% CI)	− 0.218 (− 0.384, − 0.053)
		*P* Value	*0.01*

*P* values were adjusted for gestational age, prepregnancy BMI, parity, history of delivering infants with RDS, history of macrosomia delivery and educational levels in the linear regression model

The significant *P* values (*P* < 0.05) are highlighted in italic

## Discussion

In the present study we for the first time investigated the association between the ALOX5 polymorphism rs4987105 and gestational glucose metabolism, and we observed a significant association between rs4987105 and FPG levels.

In a recent study conducted in a German cohort of 533 T2DM patients and 473 controls, Nejatian et al. reported that the minor T allele of rs4987105 was significantly less frequent in T2DM patients than in controls [OR (95% CI) = 0.7(0.54, 0.89); *P* = 0.008], indicating that the T allele was protective [29]. In agreement with their study, our analyses using both independent sample *t*-test (*P* = 0.011) and multivariate linear regression [*b* (95% CI) = − 0.218(− 0.384, − 0.053); *P* = 0.01] showed that the T allele of rs4987105 was significantly associated with lower FPG levels in the studied subjects, demonstrating that this allele may prevent the development of hyperglycemia during pregnancy.

The polymorphism rs4987105 residing in the exon 1 of *ALOX5* is a synonymous SNP close to the promoter region of this gene. Klotsman et al. showed that rs4987105 had functional impacts on the clinical responses to montelukast in asthma patients [22]. It is currently not clear how this nucleotide exchange influences the expression or activity of the enzyme. Future functional experiments are required to unravel the mechanism by which rs4987105 affects glucose metabolism.

The minor allele frequency (MAF) of rs4987105 displays ethnic variations. According to the 1000 genome data, it was lower in American (13%) and African (16%) populations, and higher in European (18%) and Asian (19%) populations. In the present study, it was 21%, which was close to the MAF in Southern Han Chinese (23%) and the MAF for the control groups reported in previous study such as Cai’s study [34] in China (18%).

It is worth noticing that BMI thresholds for increased GDM risk displays racial/ethnic distinction [35, 36]. Among American and European women, typically GDM would occur primarily among women with a higher BMI of 30 kg/m^2^ and above, whereas in Asian populations such as in our study subjects, lower BMI of only 22–23 kg/m^2^ could also confer risk of the disease. Therefore for Asian pregnant women, BMI by itself should not be considered as a reliable indicator for GDM risk prediction. Aside from obesity, it has been shown that dietary patterns and physical activity significantly influence GDM risk [37–40]. Life-style changes such as more unhealthy fast food and more sedentary activities may underlie the globally escalating prevalence of the disease. Diets such as Mediterranean Diet (MedDiet) and Dietary Approaches to Stop Hypertension (DASH) diet, as well as less sedentary activities before or during early pregnancy may be effective strategies for preventing against the development of GDM nowadays.

As far as we know, this is the first report of the association between rs4987105 of *ALOX5* and gestational glucose metabolism.

## Limitations

The main drawback of this study is the limited statistical power in only 241 patients and 139 controls. Besides, several confounding factors were not included and analyzed in this study, such as gestational weight gain, dietary and physical activity habits. Future multi-centered studies in different ethnic groups with larger sample sizes are needed to validate this finding.

## Data Availability

Data and material are available for anyone who concerns by request (email).

## Abbreviations

BMI: Body mass index
CI: Confidence interval
GDM: Gestational diabetes mellitus
MAF: Minor allele frequency
OGTT: Oral glucose tolerance test
OR: Odds ratio
PGDM: Pre-gestational diabetes mellitus
SNP: Single nucleotide polymorphism
T2DM: Type 2 diabetes mellitus
FPG: Fasting plasma glucose
ALOX5: Arachidonate 5-lipoxygenase

## References

[1] Dugas C, Perron J, Kearney M, Mercier R, Tchernof A, Marc I, Weisnagel SJ, Robitaille J (2017). Postnatal prevention of childhood obesity in offspring prenatally exposed to gestational diabetes mellitus: where are we now?. Obes Facts.

[2] Buchanan TA, Xiang A, Kjos SL, Watanabe R (2007). What is gestational diabetes?. Diabetes Care.

[3] Buchanan TA, Xiang AH (2005). Gestational diabetes mellitus. J Clin Invest..

[4] Buchanan TA, Xiang AH, Page KA (2012). Gestational diabetes mellitus: risks and management during and after pregnancy. Nat Rev Endocrinol..

[5] Alfadhli EM (2015). Gestational diabetes mellitus. Saudi Med J.

[6] Group HSCR, Metzger BE, Lowe LP, Dyer AR, Trimble ER, Chaovarindr U, Coustan DR, Hadden DR, McCance DR, Hod M, McIntyre HD, Oats JJ, Persson B, Rogers MS, Sacks DA (2008). Hyperglycemia and adverse pregnancy outcomes. N Engl J Med.

[7] Yang X, Hsu-Hage B, Zhang H, Zhang C, Zhang Y, Zhang C (2002). Women with impaired glucose tolerance during pregnancy have significantly poor pregnancy outcomes. Diabetes Care.

[8] Bellamy L, Casas JP, Hingorani AD, Williams D (2009). Type 2 diabetes mellitus after gestational diabetes: a systematic review and meta-analysis. Lancet.

[9] Hillier TA, Pedula KL, Schmidt MM, Mullen JA, Charles MA, Pettitt DJ (2007). Childhood obesity and metabolic imprinting: the ongoing effects of maternal hyperglycemia. Diabetes Care.

[10] Hauguel-de Mouzon S, Guerre-Millo M (2006). The placenta cytokine network and inflammatory signals. Placenta.

[11] Sargent IL, Borzychowski AM, Redman CW (2006). NK cells and human pregnancy–an inflammatory view. Trends Immunol.

[12] Rusterholz C, Hahn S, Holzgreve W (2007). Role of placentally produced inflammatory and regulatory cytokines in pregnancy and the etiology of preeclampsia. Semin Immunopathol..

[13] Lekva T, Norwitz ER, Aukrust P, Ueland T (2016). Impact of systemic inflammation on the progression of gestational diabetes mellitus. Curr Diab Rep..

[14] Richardson AC, Carpenter MW (2007). Inflammatory mediators in gestational diabetes mellitus. Obstet Gynecol Clin North Am.

[15] Sifnaios E, Mastorakos G, Psarra K, Panagopoulos ND, Panoulis K, Vitoratos N, Rizos D, Creatsas G (2019). Gestational diabetes and T-cell (Th1/Th2/Th17/Treg) immune profile. Vivo..

[16] Haeggstrom JZ (2018). Leukotriene biosynthetic enzymes as therapeutic targets. J Clin Invest..

[17] Filgueiras LR, Serezani CH, Jancar S (2015). Leukotriene B4 as a potential therapeutic target for the treatment of metabolic disorders. Front Immunol..

[18] Li P, Oh DY, Bandyopadhyay G, Lagakos WS, Talukdar S, Osborn O, Johnson A, Chung H, Maris M, Ofrecio JM, Taguchi S, Lu M, Olefsky JM (2015). LTB4 promotes insulin resistance in obese mice by acting on macrophages, hepatocytes and myocytes. Nat Med.

[19] Long EK, Hellberg K, Foncea R, Hertzel AV, Suttles J, Bernlohr DA (2013). Fatty acids induce leukotriene C4 synthesis in macrophages in a fatty acid binding protein-dependent manner. Biochim Biophys Acta.

[20] Mehrabian M, Schulthess FT, Nebohacova M, Castellani LW, Zhou Z, Hartiala J, Oberholzer J, Lusis AJ, Maedler K, Allayee H (2008). Identification of ALOX5 as a gene regulating adiposity and pancreatic function. Diabetologia.

[21] Schwartzman ML, Iserovich P, Gotlinger K, Bellner L, Dunn MW, Sartore M, Grazia Pertile M, Leonardi A, Sathe S, Beaton A, Trieu L, Sack R (2010). Profile of lipid and protein autacoids in diabetic vitreous correlates with the progression of diabetic retinopathy. Diabetes.

[22] Klotsman M, York TP, Pillai SG, Vargas-Irwin C, Sharma SS, van den Oord EJ, Anderson WH (2007). Pharmacogenetics of the 5-lipoxygenase biosynthetic pathway and variable clinical response to montelukast. Pharmacogenet Genomics.

[23] Li Y, Xu X, Zhang D, Cheng W, Zhang Y, Yu B, Zhang Y (2019). Genetic variation in the leukotriene pathway is associated with myocardial infarction in the Chinese population. Lipids Health Dis..

[24] Liu D, Liu L, Song Z, Hu Z, Liu J, Hou D (2017). Genetic variations of oxidative stress related genes ALOX5, ALOX5AP and MPO modulate ischemic stroke susceptibility through main effects and epistatic interactions in a chinese population. Cell Physiol Biochem.

[25] Sery O, Hlinecka L, Povova J, Bonczek O, Zeman T, Janout V, Ambroz P, Khan NA, Balcar VJ (2016). Arachidonate 5-lipoxygenase (ALOX5) gene polymorphism is associated with Alzheimer’s disease and body mass index. J Neurol Sci.

[26] Mothe-Satney I, Filloux C, Amghar H, Pons C, Bourlier V, Galitzky J, Grimaldi PA, Feral CC, Bouloumie A, Van Obberghen E, Neels JG (2012). Adipocytes secrete leukotrienes: contribution to obesity-associated inflammation and insulin resistance in mice. Diabetes.

[27] Horrillo R, Gonzalez-Periz A, Martinez-Clemente M, Lopez-Parra M, Ferre N, Titos E, Moran-Salvador E, Deulofeu R, Arroyo V, Claria J (2010). 5-lipoxygenase activating protein signals adipose tissue inflammation and lipid dysfunction in experimental obesity. J Immunol..

[28] Ying W, Wollam J, Ofrecio JM, Bandyopadhyay G, El Ouarrat D, Lee YS, Oh DY, Li P, Osborn O, Olefsky JM (2017). Adipose tissue B2 cells promote insulin resistance through leukotriene LTB4/LTB4R1 signaling. J Clin Invest..

[29] Nejatian N, Hafner AK, Shoghi F, Badenhoop K, Penna-Martinez M (2019). 5-Lipoxygenase (ALOX5): genetic susceptibility to type 2 diabetes and vitamin D effects on monocytes. J Steroid Biochem Mol Biol.

[30] Tsekmekidou XA, Kotsa KD, Tsetsos FS, Didangelos TP, Georgitsi MA, Roumeliotis AK, Panagoutsos SA, Thodis ED, Theodoridis MT, Papanas NP, Papazoglou DA, Pasadakis PS, Eustratios MS, Paschou PI, Yovos JG (2018). Assessment of association between lipoxygenase genes variants in elderly Greek population and type 2 diabetes mellitus. Diab Vasc Dis Res..

[31] Robitaille J, Grant AM (2008). The genetics of gestational diabetes mellitus: evidence for relationship with type 2 diabetes mellitus. Genet Med..

[32] Watanabe RM, Black MH, Xiang AH, Allayee H, Lawrence JM, Buchanan TA (2007). Genetics of gestational diabetes mellitus and type 2 diabetes. Diabetes Care.

[33] American Diabetes A (2014). Diagnosis and classification of diabetes mellitus. Diabetes Care.

[34] Cai C, Zhou MX, Li YP, Chen CS (2011). Association of leukotriene gene polymorphisms with response to antileukotriene treatment in patients with asthma. Zhonghua Jie He He Hu Xi Za Zhi..

[35] Hedderson M, Ehrlich S, Sridhar S, Darbinian J, Moore S, Ferrara A (2012). Racial/ethnic disparities in the prevalence of gestational diabetes mellitus by BMI. Diabetes Care..

[36] Shah A, Stotland NE, Cheng YW, Ramos GA, Caughey AB (2011). The association between body mass index and gestational diabetes mellitus varies by race/ethnicity. Am J Perinatol..

[37] Mijatovic-Vukas J, Capling L, Cheng S, Stamatakis E, Louie J, Cheung NW, Markovic T, Ross G, Senior A, Brand-Miller JC, Flood VM (2018). Associations of diet and physical activity with risk for gestational diabetes mellitus: a systematic review and meta-analysis. Nutrients..

[38] Shin D, Lee KW, Song WO (2015). Dietary patterns during pregnancy are associated with risk of gestational diabetes mellitus. Nutrients..

[39] Padmapriya N, Bernard JY, Liang S, Loy SL, Cai S, Zhe IS, Kwek K, Godfrey KM, Gluckman PD, Saw SM, Chong YS, Chan JKY, Müller-Riemenschneider F (2017). Associations of physical activity and sedentary behavior during pregnancy with gestational diabetes mellitus among Asian women in Singapore. BMC Pregnancy Childbirth..

[40] Wagnild JM, Hinshaw K, Pollard TM (2019). Associations of sedentary time and self-reported television time during pregnancy with incident gestational diabetes and plasma glucose levels in women at risk of gestational diabetes in the UK. BMC Publich Health..

